# Computational modeling of oxytocin-receptors interactions with the common marmoset *Callithrix jacchus* Pro^8^OT variant

**DOI:** 10.1590/1678-4685-GMB-2025-0058

**Published:** 2025-12-01

**Authors:** Bruno Veber, Rodrigo dos Santos Fuscaldo, Pedro Vargas-Pinilla, Bruna Oliveira Missaggia, Maria Cátira Bortolini, Paulo Augusto Netz

**Affiliations:** 1 Universidade Federal do Rio Grande do Sul, Instituto de Biociências, Programa de Pós-Graduação em Genética e Biologia Molecular (PPGBM), Departamento de Genética, Laboratório de Evolução Humana e Molecular (LEHM), Porto Alegre, RS, Brazil; 2 Universidade Federal do Rio Grande do Sul, Instituto de Química, Grupo de Química Teórica, Porto Alegre, RS, Brazil

**Keywords:** Oxytocinergic system, new world monkeys, oxytocin variant, molecular dynamics

## Abstract

The oxytocinergic system plays a crucial role in regulating physiological and behavioral processes, making it a key component of neurobiology in both humans and animals. This study utilizes computational modeling to explore the interaction between G protein-coupled receptors (GPCRs) and the *Homo sapiens* neurohormone oxytocin (Leu^8^OT), as well as, for the first time, the Pro^8^OT variant found in *Callithrix jacchus* and other New World monkeys. Pro^8^OT has been previously recognized for its functional and evolutionary significance. We performed homology modeling of receptors (OTR, VTR1a, and VTR1b) in both human and marmoset species. Additionally, cholesterol’s role in modulating receptor binding and stability was evaluated in our simulations. Our findings suggest a general pattern across primates, preserving the essential pleiotropic functions of the oxytocinergic system in regulating physiology and behavior, which align with the shared evolutionary framework of species within this order. However, some specific variations were observed, as Pro^8^OT exhibits distinct binding affinities and unique receptor interactions. Comparative analysis of human and marmoset OT-OTR complexes indicate a more stable and favorable binding environment in marmoset systems, suggesting species-specific adaptations. These results enhance our understanding of the oxytocinergic system, bridging computational models with evolutionary neurobiology and providing insights for future functional studies.

## Introduction

In placental mammals, oxytocin (OT) and its paralog arginine-vasopressin (VT) are considered key neurohormones in the oxytocinergic system precisely because they act in the central nervous system, where they are produced, modulating complex social behaviors (parental care, pair bond and stress control, among others), as well as in the peripheral, related to basic physiological processes such as uterine contraction and ejection of breast milk (Lucion and Bortolini, 2014; Paré et al., 2016; French et al., 2016; Cavanaugh et al., 2018; Freeman and Bales, 2018). Until a few years ago, it was thought that all placental mammals shared the same nine-amino acid sequence of OT (Cys-Tyr-Ile-Gln-Asn-Cys-Pro-Leu-Gly, here called Leu^8^OT), known since the 1950s (Du Vigneaud et al., 1953). The preservation of the OT sequence in all placental mammals has been challenged, particularly among New World monkeys (NWm; Platyrrhini parvordem). Variants of OT, such as Pro^8^OT and Val^3^Pro^8^OT, have been identified in different primate families. In these cases, the leucine and isoleucine residues were substituted with proline and valine, respectively, at the eighth and third positions of the neurohormone’s amino acid chain. For instance, Pro^8^OT, a derived OT form, is present in species from the Cebidae, Callitrichidae, and Atelidae families of the Platyrrhini clade, while Val^3^Pro^8^OT is found only in *Saguinus*, a genus belonging to the Callitrichidae family. The Ala^8^OT form was observed in *Cacajao melanocephalus* and *Chiropotes utahickae*, Thr^8^OT in *Chiropotes albinasus*, both genera of the Pitheciidae family (Vargas-Pinilla et al., 2015). Other studies revealed the variants Phe^2^OT in *Alouatta caraya* (Ren et al., 2015), Val^3^OT in *Alouatta seniculus*, species of the Atelidae family (Mustoe et al., 2018), and Val^3^OT in *Alouatta sara*, while other *Alouatta* species presented the ancestral form Leu^8^OT (Campbell and Cortés-Ortiz, 2021). Pro^8^OT in *Ateles fusciceps* was also reported (Campbell and Cortés-Ortiz, 2021). New findings and the extent of these OT variation across Primates order can be seen in Vargas-Pinilla *et al.* (2024). Also, it is noteworthy that VT does not vary in these same species.

Taken together, these findings strongly indicate taxon-specific variations in the OT molecule among Platyrrhini species, and these could have significant implications for our understanding of oxytocinergic involvement in social and reproductive behaviors, given its well-established importance in bonding, maternal behaviors, and uterine contractions in placental mammals. For instance, our research group has demonstrated that at least the Pro^8^OT and *Saguinus* Val^3^Pro^8^OT variants have played a key role in the successful evolutionary trajectory of tamarins and marmosets (Callitrichidae family), as they exhibit rare reproductive behavior patterns compared to other mammals, including other primates. Therefore, these variants would be part of the exclusive genetic repertoire that allowed the emergence of adaptive behaviors, such as social monogamy and the direct care of fathers and other males to infants. These animals are also characterized by having a small size and twin births, which makes social monogamy and the presence of tolerant and careful males key elements in increasing the adaptive value of the twins (Vargas-Pinilla et al., 2015). 

Activation of OT occurs through interaction with the oxytocin receptor (OTR), a plasma membrane receptor that promotes cell signaling by coupling to G proteins, specifically G protein-coupled receptors (GPCRs). OTR signals, primarily but not exclusively, via Gq (α subunit), which activates membrane phospholipase C (PLC), leading to the production of diacylglycerol (DAG) and inositol triphosphate (IP3). These, in turn, lead to increased intracellular levels of calcium. DAG and calcium are classic activators of protein kinase C (PKC). All these signaling pathways are involved in a wide range of normal biological functions, as well as in pathologies when the expected function does not occur (Preininger and Hamm, 2004; Dorsam and Gutkind, 2007; Lambert, 2008). The Gq-coupled receptor, in turn, forms a complex with β-arrestins 1 and 2 capable of desensitizing and internalizing the GPCR. This set of actions can be considered canonical, as it is relatively well-known. 

It is also known that OT interacts with VT receptors (the GPCRs VTR1a, VTR1b, VTR2) as well as VT with OTR, but with different levels of affinity. For instance, VT has a similar affinity for OTR, VTR1a, and VTR1b, whereas OT may have a higher affinity for its canonical receptor, OTR, than for VTR1a and VTR1b (Song and Albers, 2018). This general lack of receptor selectivity is not surprising, considering that these receptors have a high degree of structural homology (Song and Albers, 2018). However, both OT and VT receptors are rarely found in the same synaptic regions; the likelihood of cross-talk occurring after the synaptic release of these neuropeptides would be low, except in cases where these neuropeptides diffuse beyond the synaptic boundaries. As a result, the nonsynaptic release of OT or VT, which may be substantial under certain physiological conditions, may activate a broad array of OT and VT receptors beyond synaptic boundaries (Song and Albers, 2018), potentially modulating canonical G protein-coupled signaling pathways within the oxytocinergic system, but not limited to it.

Unlike other receptors that modulate complex social behaviors and are also expressed in the brain, VTR2 is synthesized in the kidneys, acting solely in physiological processes such as the renal regulation of water and electrolyte homeostasis (Lolait et al., 1992; Fam et al., 2023).

Moreover, it was possible to identify that some of the OT derived forms (*e.g*., Pro^8^OT and *Saguinus* Val^3^Pro^8^OT) were coevolving with the specific OTRs and other oxytocinergic molecules in the corresponding species (Vargas-Pinilla et al., 2015). 

The pioneering study by Chini et al. (1995) highlighted that positions 3 and 8 of oxytocin (OT) are critical for interactions that enhance the peptide’s affinity for the oxytocin receptor (OTR). Posteriorly, our research group (Parreiras-e-Silva et al., 2017) performed *in vitro* and *in vivo* studies comparing Pro^8^OT and Val^3^Pro^8^OT with Leu^8^OT and VT. These two derived OT forms were initially synthesized and then tested with various parameters, considering their interaction with the commercially available human receptors OTR and VTR1a, which are considered the most important modulators of prosocial behaviors within the OT-VT system. We demonstrated that Pro^8^OT and *Saguinus* Val^3^Pro^8^OT are equally efficient agonists, when compared to Leu^8^OT, in mediating G protein-dependent pathways, particularly the Gq/11 isoforms, and in calcium mobilization, but that they have a reduced capacity for recruitment of β-arrestins 1 and 2, decreasing or even preventing the internalization of human OTR, therefore having a possible and relevant impact on the desensitization of the entire system (Parreiras-e-Silva *et al.*, 2017). In other words, these variants enable the maintenance of high OTR levels in the plasma membrane, thereby amplifying the Gq-dependent signaling pathway. *Saguinus* Val^3^Pro^8^OT also showed reduced recruitment of β-arrestins after interaction with the human VTR1a receptor, suggesting a differentiated action with at least one of the VT receptors. It is important to note that our experiments performed in Parreiras-e-Silva *et al.* (2017) used NWm ligands in combination with human receptors; therefore, the results should be interpreted with caution and considered within a general, non-species-specific framework.

For the *in vivo* tests, the synthesized forms Pro^8^OT and Val^3^Pro^8^OT were administered via intranasal spray in 48 pairs of WTG rats (*Rattus norvegicus*). According to well-established parameters and protocols for behavioral studies, an increase in maternal behavior was also observed, as well as unusual paternal care in rats, as measured by specific tests (Parreiras-e-Silva et al., 2017). For instance, when male rats received Val^3^Pro^8^OT treatment, which exhibited the most pronounced pharmacological alterations among the derived OT forms tested, they initiated contact with their offspring more rapidly than rats subjected to other intranasal treatments. This observation suggests that treatments involving *Saguinus* Val^3^Pro^8^OT had a notable effect in increasing the responsiveness of male rats to engage and interact with their offspring (Parreiras-e-Silva *et al.*, 2017).

With these studies, Parreiras-e-Silva et al. (2017) demonstrated for the first time a natural system with possible adaptive relevance, where a particular ligand (Pro^8^OT or *Saguinus* Val^3^Pro^8^OT) can differentially modulate intracellular signaling through the same GPCR (agonism with functional selectivity or biased agonists) (Costa-Neto et al., 2016), a relatively new concept relevant to several areas of interest, from basic academic/scientific to pharmacology given the importance of GPCRs in the context of the pharmaceutical industry (Wright and Bouvier, 2021). In other words, GPCR signaling can proceed through multiple pathways, such as G protein-mediated calcium mobilization or β-arrestin recruitment. However, in cases of functionally selective agonism, such as with Pro^8^OT and *Saguinus* Val^3^Pro^8^OT, one pathway may be preferentially activated (Parreiras-e-Silva *et al.*, 2017), enabling the targeted modulation of cellular responses while maintaining the physiological integrity of the other signaling routes.

It is well known that cholesterol (CLR, a sterol-like type of lipid) constitutes about 30% of all animal cell membranes. The primary function of CLR is structural, as it regulates membrane fluidity. CLR also interacts with many membrane proteins, including GPCRs (*e.g*., human OTR; Klein et al., 1995). Lipids are known to influence bilayer properties such as thickness, curvature, and surface tension (Dawaliby et al., 2016). Nevertheless, some authors have also provided experimental evidence that certain lipids, such as phospholipids, can modulate GPCR activity even in the absence of a lipid bilayer (Dawaliby *et al.*, 2016). This interaction alters receptor properties related to ligand binding, receptor activation, and signal transduction, and is therefore considered a form of allosteric modulation of GPCRs (Jakubík and El-Fakahany, 2021). Furthermore, CLR is also known to directly modulate the function and stability of OTR in a highly specific manner, independently of membrane fluidity (Gimpl et al., 2000). In their *in vitro* assays, the authors also showed that the addition of CLR protected OTR from thermal denaturation and partially restored its high-affinity binding to OT.

However, this promising field remains challenging due to the complexity of these interactions and the taxon-specific variations in primates that need to be carefully considered. To contribute to this area, we conducted advanced computational simulations and molecular modeling to investigate the interactions between Leu^8^OT, Pro^8^OT, and the allosteric modulator CLR. Additionally, we investigated the dynamics of cross-talk between these neurohormones and non-canonical VT receptors. By utilizing computational simulations and molecular modeling, we aim to elucidate the molecular mechanisms underlying the dynamics of the oxytocinergic system, with potential implications for adaptive phenotypes in primate species. The present work also provides a foundation for future research on the functional and evolutionary consequences of the extraordinary variability of the oxytocinergic system across primate species.

## Material and Methods

### Ligand construction

The ancestral OT (Leu^8^OT), derived OT (Pro^8^OT) and cholesterol structures were constructed from sequence, using the ChemBioDraw Ultra 14.0 software (Chemdraw RRID:SCR_016768). The geometries were optimized with the MOPAC (Stewart, 2016) program using the PM6 semi-empirical method (Stewart, 2007).

### Homology modeling for receptors

The construction of homology models of the forms of OTR, VTR1a and VTR1b present in primates was made in the Modeller program on the MPI Bioinformatics Toolkit web server (Šali et al., 1995; Zimmermann et al., 2018; Gabler et al., 2020). The sequences of the coding region of the *OTR*, *VTR1a,* and *VTR1b* genes for the species of interest are described by our research group and other researchers (Ren et al., 2014; Vargas-Pinilla et al., 2015; Fam et al., 2019). More, recently, the crystal structure of human OTR in complex with an antagonist and with cholesterol has been described. The reference structure for the homology models was the OTR crystal structure (6TPK) available for public access on the ProteinDataBank website (10.2210/pdb6TPK/pdb) (Waltenspühl et al., 2020). The homology models were validated using Ramachandran plot and G-factor statistics from PROCHECK analyses (Laskowski et al., 1993).

### Molecular docking

The structures of the ligands and receptors were prepared using AutoDockTools (Morris et al., 2009), and molecular docking was carried out with the AutoDock Vina program (Trott and Olson, 2010) to estimate the ideal initial position of the ligands in the corresponding receptor structures for molecular dynamics simulations. The docking grid was centered on the receptor-binding site, with the exception of cholesterol docking, which was centered on the cholesterol-binding site. Cholesterol docking was performed using a blind docking approach to ensure coverage of the entire surface of the molecule. The exhaustiveness was optimized until the docking results could be reliably reproduced in triplicate, with a range between 8 and 12. Only the flexibility of the ligands was considered, while the receptor structure remained rigid during the simulations. For each docking run, the pose with the most negative free energy (docking score) was analyzed, focusing on its position and interaction patterns. The experiments included both canonical complexes (ligand and receptor from the same species and in their natural forms) and cross-docking assays involving non-canonical combinations of ligands and receptors.

### Molecular dynamics simulation

The GROMACS software (Abraham et al., 2015) was utilized to conduct molecular dynamics simulations, producing triplicate trajectories of the receptor-ligand complexes. These trajectories were analyzed to ascertain various parameters, including the number of hydrogen bonds, the stability of the complexes, and the mapping of molecular interactions. The protein topology was defined using parameters from the AMBER ff99sb force field (Lindorff-Larsen et al., 2010), while the ligand topologies were constructed with parameters compatible with this force field using the ACPYPE program (Silva and Vranken, 2012). The systems were solvated with TIP3P-type water molecules (Jorgensen et al., 1983) and were contained in dodecahedron-shaped boxes. Initially, the systems underwent energy minimization and equilibration in both the NVT and NPT ensembles. The energy minimization process was carried out using the steepest gradient algorithm for 5000 cycles. Long-range electrostatic interactions were calculated with the Particle Mesh Ewald algorithm (Darden et al., 1993), employing a cutoff radius of 8.0 Å. The time step for the simulation was set to 2.0 fs, and trajectories were recorded every 10 ps over a total simulation duration of 100 ns. This simulation time was selected based on the premise that most events of biochemical interest, such as functionally important structural rearrangements, typically occur on nanosecond to microsecond timescales (Hollingsworth and Dror, 2018). Receptor-ligand interaction diagrams were generated using BIOVIA Discovery Studio Visualizer (BIOVIA, 2020). For the structural analysis of each system, the root mean square deviation (RMSD), root mean square fluctuation (RMSF), and hydrogen bond (H-bond) distribution were computed. In defining a hydrogen bond, the criteria used were that the distance between the two electronegative atoms, referred to as D and A, must be ≤ 0.35 nm and the angle H-D-A must be ≤ 30 °.

### Binding free energy assays

The binding free energy between the ligand and protein was estimated using the Molecular Mechanics Poisson-Boltzmann Surface Area (MM/PBSA) method, as described in the literature (Srinivasan et al., 1998; Massova and Kollman, 2000; Miller et al., 2012; Genheden and Ryde, 2015). The calculations were performed with the GROMACS-compatible program “gmx_MMPBSA” (Valdés-Tresanco et al., 2021). In the MM/PBSA approach, the free energy of binding between the ligand and receptor is calculated as a sum of contributions from bonded and non-bonded intramolecular and intermolecular interactions, as well as a solvation free energy. This last term is composed of polar and non-polar solvation, where the former is calculated by solving the Poisson-Boltzmann equation and the latter is estimated as a function of solvent-accessible surface area. MM/PBSA parameters for polar/solvation calculations were set as follows: internal dielectric constant, 1; external dielectric constant, 80; probe radius, 1.4; cavity surface tension, 0.0378; cavity offset, -0.5692; and the remaining parameters in their default values.

### Amino acid comparison

A comparative amino acid analysis was conducted on three receptor proteins, OT, VTR1a, and VTR1b, in *Homo sapiens* and *Callithrix jacchus*. The objective was to identify and compare amino acid positions critical for interaction with Leu^8^OT in humans and Pro^8^OT in *Callithrix jacchus*.

For OTR, the sequences XP_017820536.1 for *Callithrix jacchus* and NP_000907.2 for *Homo sapiens* were used, with UniProt (https://www.uniprot.org/) (UniProt Consortium, 2018) codes F7ILY7_CALJA and P30559, respectively.

In the case of VTR1a, the analyzed sequences were XP_009002391.1 for *Callithrix jacchus* and NP_000697.1 for *Homo sapiens*, with UniProt codes U3BDH7_CALJA and P37288, respectively.

For VTR1b, the sequences XP_035136701.2 for *Callithrix jacchus* and NP_000698.1 for *Homo sapiens* were used, with UniProt codes A0A2R8MAU6_CALJA and P47901, respectively.

Sequence alignment was generated by NCBI (https://www.ncbi.nlm.nih.gov/) (O’Leary et al., 2016) using all available primate sequences in the database. We utilized the sites of most significant interaction between the oxytocins and their receptors, as identified by the PBSA method (with cholesterol), to compare the interaction homology between the orthologous proteins of the two species. Comparisons of homologous positions with different amino acids between species and the critical positions for the respective receptor-hormone interactions were performed using Python scripts (Van Rossum and Drake Jr., 1995). Images of the respective receptor structures for each species were generated using Protter (https://wlab.ethz.ch/protter/start/) (Omasits et al., 2014).

## Results and Discussion

### Homology modeling for receptors

Previous studies verifying the structure of OTR with the ligand were conducted using an OTR model based on a different structural coverage prediction approach (Koehbach et al., 2013; Antobreh *et al.*, 2018; Miladinova et al., 2020). In these studies, the predicted models were built using a common structure of a GPCR and computationally analyzed with the common ligand Leu^8^OT. Herein, we employed for homology modelling the OTR, VTR1a and VTR1b, the template of the crystal structure of *Homo sapiens* OTR described (PDB ID: 6TPK) (Waltenspühl et al., 2020) because of sequence identity, resolution and query cover. The OTR, VTR1a and VTR1b primary sequences of *Homo sapiens* and *Callithrix jacchus,* the common marmoset characterized by the Pro^8^OT presence, paternal care, social monogamy and twin births, were retrieved from the NCBI database. This dataset was used for homology modeling of the receptors using the MODELLER software package. A total of six receptor structures were produced by modeling in our work. All structures can be made available on demand.

The analysis of the Ramachandran plots (Table 1) provides a stereochemical evaluation of the backbone ψ and φ dihedral angles in our models. Based on a reference dataset of 118 high-resolution crystal structures (≤ 2.0 Å), it is recommended that the cumulative percentage of residues in the ‘Additionally Allowed’, ‘Generously Allowed’, and ‘Disallowed’ regions should not exceed 20%, which is indicative of a good-quality model (Laskowski et al., 1993). In our models, these regions remain below this threshold. The residues in the most favored regions for the human OTR and the marmoset *Callithrix jacchus* VTR1a and VTR1b account for 89.3%, 89.5%, and 88.6%, respectively, further supporting the sound quality of these structures. The G-factors provide a measure of how unusual, or out-of-the-ordinary, a property is, considering the torsion angles and bond lengths in the main chain. Values below -0.5 are considered uncommon, and those below -1.0 are highly unusual. None of the models exceed this limit, indicating good consistency in the constructed models.

**Table 1-:** Analysis from Ramachandran plots and PROCHECK G-factor^a^ findings.

Model	Most favored regions	Additionally allowed regions	Generously allowed regions	Disallowed regions	G-factor
*Homo sapiens*					
OTR	89.3%	7.6%	2.1%	1.0%	-0.12
VTR1a	91.4%	7.0%	1.3%	0.3%	-0.04
VTR1b	90.0%	8.1%	0.6%	1.3%	-0.09
Marmoset *Callithrix jacchus*					
OTR	90.7%	8.6%	0.7%	0.0%	-0.11
VTR1a	89.5%	7.0%	3.2%	0.3%	-0.10
VTR1b	88.6%	9.1%	1.0%	1.3%	-0.15

^a^ The G-factor from PROCHECK is a measure used to assess the stereochemical quality of protein structures. Values greater than -0.5, such as those ranging from -0.04 to -0.15 observed in the sixth column of the table, are considered indicative of well-refined structures.

### Molecular docking

Molecular docking was conducted for all target receptors and their respective ligands. To predict the most likely configuration of each ligand-receptor complex, initial blind docking was performed using a grid box encompassing the entire molecular complex in each set. Subsequently, the grid box was centered within the binding pocket to carry out precise molecular docking. The results revealed the most stable conformation for each complex. Triplicates of OTR, VTR1a, and VTR1b complexes, along with their respective ligands, consistently adopted a common conformation. These results indicate strong interactions (Table 2), surpassing even the potentially canonical receptor (OTR), as the interaction remains robust with the VT-canonical receptors, VTR1a and VTR1b. Cross-docking results were generally less favorable, except for the complex involving marmoset Pro^8^OT with marmoset VTR1a and human OTR. In competition binding assays Taylor et al. (2018) tested Ca²⁺ signaling in OTRs from four primates, two Catarrhini (*Homo sapiens, Macaca mulatta*) and two Platyrrhini (*Callithrix jacchus, Callicebus cupreus*), stimulated with Leu⁸OT or Pro⁸OT. Although they expected receptors to respond best to the species’ own OT variant, results did not fully support high specificity. In *C. jacchus*, Pro⁸OT was slightly more potent, as expected. Interestingly, Pro⁸OT also exhibited stronger binding than Leu⁸OT even in Leu⁸OT-producing species. Since both ligands elicited similar Ca²⁺ responses, the authors suggested that downstream signaling mechanisms, together with the lower likelihood of the flexible tail of Leu⁸OT adopting the optimal conformation for receptor activation, unlike the naturally rigid structure of Pro⁸OT, also play an important role (Taylor *et al.*, 2018).

**Table 2-:** Affinity scores from molecular docking of oxytocin-receptor complexes in human and marmoset: OT-OTR, OT-VTR1a, and OT-VTR1b.

Ligand	Receptor	Affinity (kJ/mol)
Leu^8^OT *Homo sapiens*	***Human OTR***	**-46.48**
	Marmoset OTR	-40.60
	**Human VTR1a**	**-38.08**
	Marmoset VTR1a	-36.84
	**Human VTR1b**	**-44.39**
	Marmoset VTR1b	-39.27
Pro^8^OT Marmoset, *Callithrix jacchus*	Human OTR	-42.23
	***Marmoset OTR***	**-41.04**
	Human VTR1a	-39.27
	**Marmoset VTR1a**	**-37.66**
	Human VTR1b	-40.60
	**Marmoset VTR1b**	**-44.81**

In *italics* and **bold** for canonical complexes (ligand and receptor from the same species and in their natural forms). In **bold** only for non-canonical cognate complexes (ligand native to the species interacting with a different receptor from the same species). No highlight for non-cognate complexes (ligand from another species).

The results of molecular docking (Table 2) showed negative affinity scores for all complexes, indicating favorable interactions, with values ranging from -36.84 kJ/mol to -46.48 kJ/mol. Simulations were performed with OT as the ligand, even for VTR1a and VTR1b, whose canonical hormone is VT. Canonical complexes exhibited the highest affinities for each species: *H. sapiens* Leu⁸OT-OTR (-46.48) and *C. jacchus* Pro⁸OT-OTR (-41.04). Among cognate complexes, *H. sapiens* Leu⁸OT-VTR1b (-44.39) and *C. jacchus* Pro⁸OT-VTR1b (-44.81) showed notably high affinities, with the latter being the second strongest interaction overall. VTR1a consistently displayed the lowest affinities in both species, regardless of ligand. Overall, OTR showed the greatest gain in affinity when paired with its canonical ligand (Leu⁸OT in humans, Pro⁸OT in marmosets), while VTR1b in *C. jacchus* exhibited unexpectedly high affinity for Pro⁸OT, suggesting that, despite being a VT receptor, it retains a strong capacity to bind OT in this species.

When these interaction values are compared with those reported for rats and mice (both of which possess the ancestral form Leu⁸OT, identical to humans) by Song and Albers (2018) for their respective cognate receptors OTR, VTR1a, and VTR1b, the canonical OTR complexes in humans and marmosets show lower affinity than in rodents, whereas values for VTR1a and VTR1b are closer to, or slightly higher than, those observed in rodents. Specifically, in rats the affinities were -52.89, -38.84, and -33.51 kJ/mol, and in mice -55.10, -32.10, and -31.85 kJ/mol for OTR, VTR1a, and VTR1b, respectively.

Interestingly, a pattern preserved over ~70 million years of evolution, separating the human and mouse lineages, appears to have changed with the neurohormone substitution at position 8 (Leu > Pro), observed in some clades of Platyrrhini (Vargas-Pinilla et al., 2024). This missense mutation potentially increases the affinity of neurohormone Pro^8^OT with the non-canonical receptor VTR1b in *Callithrix jacchus* (-44.81; Table 2). Biological interpretation and experimental validation are essential to fully elucidate this intriguing finding. Therefore, these results highlight the importance of ligand-receptor specificity, while also underscoring the potential for cross-talk interactions, a phenomenon previously emphasized particularly in peripheral organs (Song and Albers, 2018).

**Figure 1 -:**
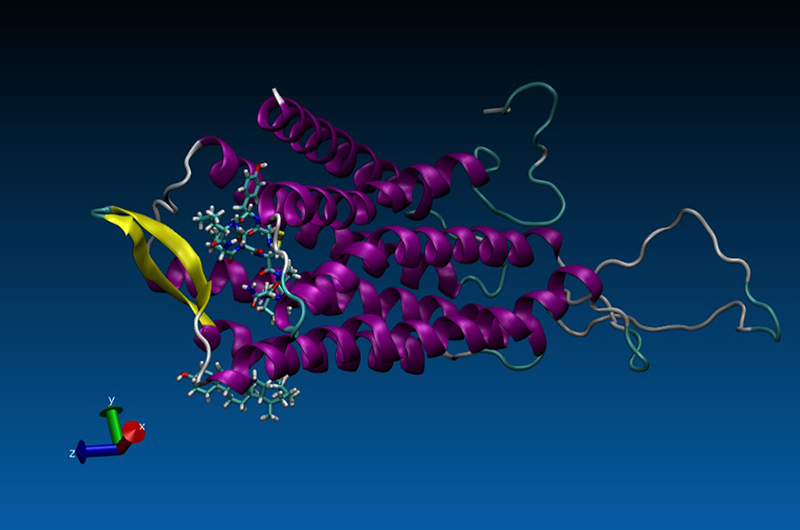
The complex structure of Homo sapiens oxytocin (OT), the oxytocin receptor (OTR) and cholesterol. The OT structure is located inside the OTR cavity, while the cholesterol structure is located outside OTR structure.

The best-known complex structure of *Homo sapiens* OT, OTR, and cholesterol (CLR) can be seen in Figure 1 and Figure S1, while the docking results with target receptors from *Homo sapiens* and *Callithrix jacchus* are presented in Table 3. The affinity values for humans are slightly more negative compared to those for marmosets (Table 3).

**Table 3 -:** Affinity between the cholesterol molecule (CLR) and receptors from human (*Homo sapiens*) and marmoset (*Callithrix jacchus*).

Species	Complex	Affinity (kJ/mol)
*Homo sapiens*	CLR+OTR	-35.99
	CLR+VTR1a	-38.08
	CLR+VTR1b	-38.08
Marmoset *Callithrix jacchus*	CLR+OTR	-34.35
	CLR+VTR1a	-34.76
	CLR+VTR1b	-34.76

There are robust evidences that CLR has allosteric modulations capabilities by shifting the receptor to a high-affinity state (Gimpl et al., 1997, 2000; Gimpl and Fahrenholz, 2002). Furthermore, a study showed that CLR may directly influence the polar network of the ligand-binding pocket through tyrosine (Y) residue at position 200 of human OTR, forming a hydrogen bond with Q171, leaving the cavity in an ideal state for ligand recognition (Waltenspühl et al., 2020). This context is preserved in *Callithrix jacchus* OTR, since a ligation with CLR is predicted at same position Y200 (Figures S1Aand S1D). For other four complexes the interaction between a tyrosine (Y) residue and CLR is predicted too (Figures S1B, S1C, S1E and S1F). It is noteworthy that the same residue is found at different positions due to variations in the receptor sequences. Precisely, the Y200 in OTR receptors (both human and marmoset) corresponds to position 216 in VTR1a receptors (both human and marmoset), position 199 in human VTR1b, and position 220 in marmoset VTR1b. Interestingly, our study revealed two additional residues involved in typical interactions. The first is an alanine located immediately before the tyrosine residue in these sequences. This alanine undergoes an amino acid substitution to valine in marmoset VTR1b but still interacts with cholesterol. The second residue is a tryptophan positioned three positions after the tyrosine residue in the local arrangement.

### Molecular dynamics simulations

The molecular dynamics simulations were run at 310 K in a water environment with and without a cholesterol molecule. The simulations were carried out in three replicas with 100 ns long simulation runs, to observe if there were significant conformational changes during the trajectory in both cases (with and without cholesterol). The following parameters were calculated: root mean square deviation, root mean square fluctuations, hydrogen bonds and interaction free energies. A map of receptor-ligand interactions was generated on a 2D diagram at BIOVIA Discovery Studio Visualizer. 

The structural stability of the simulated complexes was observed through the root mean square deviation (RMSD) analysis (Figure S2). The analysis was performed by disregarding the C-Term portion and the ICL3 loop, due to the high mobility of these structural parts, which can destabilize the recorded values. The RMSD values of human and marmoset complexes without cholesterol were all between 0.3 nm and 0.7 (Figures S2A, S2B, S2D, S2E and S2F), except for human Leu^8^OT-VTR1b (Figure S2C) that show a variation between 0.6 nm and 1 nm in our triplicate runs. Furthermore, the human and marmoset OT-OTR complexes with cholesterol had the RMSD values between 0.5 nm and 0.8 nm (Figures S2G and S2J), the human and marmoset OT-VTR1a complexes had values between 0.75 nm and 1 nm (Figures S2H and S2K), the human Leu^8^OT-VTR1b complex had values between 0.6 nm and 0.8 nm (Figure S2I) and the marmoset Pro^8^OT-VTR1b complex had values between 0.5 nm and 0.6 nm (Figure S2L). Considering the overall RMSD profiles, we can conclude that, except for one of the replicates of human Leu^8^OT-VTR1b, the systems reached structural equilibrium, and the sets of three 100-ns-long simulations for each system are reliable for sampling the relevant properties. This simulation timescale is consistent with the duration at which most events of biochemical interest, such as functionally relevant conformational changes in proteins, typically occur (Hollingsworth and Dror, 2018).

The structural fluctuations of each amino acid were monitored by RMSF plots as a function of residue number to observe the flexibility of the complexes during the trajectory (Figure S3). Higher RMSF values reflect increased atomic fluctuations, which often correspond to flexible structural elements like loops or indicate reduced structural stability in binding regions due to weaker intermolecular interactions. Portraying the individual residues sites, greater fluctuations can be seen at the amino acids 230-260 at following systems: Leu^8^OT-OTR-human (Figure S3A), Leu^8^OT-VTR1b-human (Figure S3C), Pro^8^OT-OTR-marmoset (Figure S3D), CLR-Leu^8^OT-OTR-human (Figure S3G), CLR-Leu^8^OT-VTR1b-human (Figure S3I), CLR-Pro^8^OT-OTR-marmoset (Figure S3J). All the other systems presented greater fluctuations at the amino acids 240-280. Furthermore, other fluctuations can be observed in the graphics, every increase at the value matches with all the six loops of the GPCR protein, the N-terminal portion, and accentuated mobility of C-terminal portions.

The number of hydrogen bonds between OTs and the receptors were calculated along with the simulations. Obtaining a large number of hydrogen bonds during the trajectory indicates a more stable conformation and strongest binding between compounds. As observed during simulations (Figure S4), there exist less hydrogen bonds for the human Leu^8^OT-VTR1b complex than all other human and *Callithrix jacchus* complexes. Taking an average over the simulation period, it is seen that the complexes *Homo sapiens* Leu^8^OT-OTR, CLR-Leu^8^OT-OTR, Leu^8^OT-VTR1a, CLR-Leu^8^OT-VTR1a, and CLR-Leu^8^OT-VTR1b, and marmoset *Callithrix jacchus* Pro^8^OT-OTR, CLR-Pro^8^OT-OTR, CLR-Pro^8^OT-VTR1a, Pro^8^OT-VTR1b and CLR-Pro^8^OT-VTR1b form two hydrogen bonds, while human Leu^8^OT-VTR1b forming only one hydrogen bond and for marmoset Pro^8^OT-VTR1a forming 3 hydrogen bonds.

Significant interactions, including hydrophobic interactions, deserve our attention. We will delve into these interactions through interaction maps. It is important to note that an interaction map provides a static snapshot of a dynamic process, where interactions form and break continuously within a specific biological context. Therefore, conducting these analyses in triplicate is essential. Given the numerous hydrogen bond interactions across multiple systems and replicates, we have emphasized the interactions that consistently appeared most frequently.

In the present study, we have provided detailed information on the residues and types of interactions between receptors and their corresponding ligands, as shown in Tables S3and S4. We observed four main types of interactions: classical hydrogen bonds, non-classical hydrogen bonds (including carbon-hydrogen bonds and pi-donor hydrogen bonds), hydrophobic interactions (such as alkyl, pi-alkyl, pi-sigma, pi-pi stacked, and pi-pi T-shaped), and sulfur interactions (pi-sulfur).

In the human OTR (Table S3), both with and without cholesterol, we can observe the same tryptophan residue at position 99 (Trp99) engaging in a hydrophobic interaction with human Leu^8^OT. In the human VTR1a, both with and without cholesterol, two residues consistently interact: a glutamine at position 311 (Gln311) forms a conventional hydrogen bond, and an isoleucine (Ile330) engages in a hydrophobic interaction with the ligand. In human VTR1b, both with and without cholesterol, we observed a conventional hydrogen bond involving the Ala188 residue. 

In the marmoset OTR both with and without cholesterol (Table S5), we can observe different interactions, including conventional hydrogen bonds and hydrophobic interactions, at the Trp99 residue. Multiple interactions can be visualized in the marmoset VTR1a, both with and without cholesterol: Gln108, Gln131, and Gln311 form conventional hydrogen bonds with marmoset Pro^8^OT, while Val217 and Ile330 engage in hydrophobic interactions. Furthermore, in the case of marmoset VTR1b, both with and without cholesterol, a conventional hydrogen bond involving the Trp115 residue with the ligand can be observed. Since interaction maps provide a qualitative description of interactions, we supplemented our analysis with a more quantitative approach using gmx mmpbsa analysis. 

As a note of caution, we acknowledge that the lipid bilayer was not explicitly included in the present work, which may be seen as a limitation. Given the scope and scale of the systems analyzed, 12 in total, each simulated in triplicate, this choice allowed us to focus on comparative structural features across conditions. Importantly, the relatively low mobility observed in the transmembrane regions, as indicated by the RMSF profiles (see Figures S3 and S8-S10), suggests that the absence of the membrane is unlikely to have significantly affected the main conclusions. Moreover, Dawaliby et al. (2016) demonstrated that GPCR activity can be modulated by direct lipid interactions even in the absence of a bilayer, which supports the hypothesis that, despite the absence of an explicit membrane, our results may still offer novel and relevant insights into species-specific receptor-ligand interactions and the allosteric role of CLR. Although the modulatory effect of CLR on GPCRs has been previously demonstrated, it has not yet been explored in the context of non-human primates with such structurally divergent ligands as those analyzed here, which may help explain the differential CLR effects observed in the MM/PBSA analyses. Nevertheless, incorporating the complete membrane environment remains a critical and logical next step, and is foreseen as a continuation of the present study.

### Binding free energy assays

Binding free energy was estimated for all complexes using the MM/PBSA method. Interaction energies, including Van der Waals energy, electrostatic energy, electrostatic solvation free energy evaluated using the Poisson-Boltzmann equation, the nonpolar component of solvation energy, gas-phase energy, solvation free energy, and binding energy, were calculated over a 100 ns trajectory and are tabulated in Tables S1, S2 and S4.

The Molecular Mechanics/Poisson-Boltzmann Surface Area (MM/PBSA) analysis offers insights into the energy changes associated with the interactions between human and marmoset OTs and their cognate receptors (OTR, VTR1a, and VTR1b). The average binding free energy (ΔG_TOTAL_) values measured by MM/PBSA for human complexes range from -37.9±4.8 kJ mol^-1^ (Leu^8^OT-VTR1b) to -56.6±3.9 kJ mol^-1^ (CLR-Leu^8^OT-VTR1a), while for marmoset complexes, they range from -54.4±2.9 kJ mol^-1^ (Pro^8^OT-VTR1b) to -64.7±4.2 kJ mol^-1^ (Pro^8^OT-VTR1a) (Table 4). There is a consistent increase in the strength of Van der Waals interactions (more negative values) in marmoset complexes compared to human complexes, resulting in a higher binding affinity (Table 4). On the other hand, there is almost no difference in energy associated with the surfaces, indicating a consistent interaction pattern across all tested complexes. But, interestingly, if we compared complexes with cholesterol *versus* without it (*e.g*., human Leu^8^OT-OTR *versus* human CLR- Leu^8^OT-OTR) the ΔG_TOTAL_ in 4 of the 6 systems with cholesterol is more negative, indicating a more stable and more favorable binding environment between the neurohormone and the receptors, with the exception of human Leu^8^OT-OTR *versus* human CLR-Leu^8^OT-OTR and marmoset Pro^8^OT-VTR1a *versus* marmoset CLR-Pro^8^OT-VTR1a.

**Table 4-:** Average Binding Free Energy MM/PBSA (in kJ mol^-1^) computations for complexes of *Homo sapiens* and marmoset (*Callithrix jacchus*) oxytocins (OTs) with their cognate receptors, both in the presence and absence of cholesterol.

Organism	System	ΔE_VDWAALS_		ΔE_EL_		ΔE_PB_		ΔE_SURF_		ΔG_GAS_		ΔG_SOLV_		ΔG_TOTAL_	
**Human**	**Leu^8^OT-OTR**	-88.3	±2	52.0	±21.3	-18.9	±0.6	-3.0	±9.7	-36.3	±19.9	-15.2	±20.7	-51.6	±1
	**Leu^8^OT-VTR1a**	-91.9	±6.9	-43.0	±29.9	86.9	±30.7	-8.3	±0.3	-134.9	±35.5	78.6	±30.5	-56.3	±6.3
	**Leu^8^OT-VTR1b**	-70.4	±5.5	21.4	±27.2	18.3	±25.5	-7.2	±0.2	-49.0	±27	11.2	±25.6	-37.9	±4.8
	**CLR-Leu^8^OT-OTR**	-86.2	±6.4	37.0	±0.9	7.6	±1.4	-8.4	±0.4	-49.3	±6.8	-0.7	±1.1	-50.0	±6
	**CLR-Leu^8^OT-VTR1a**	-90.5	±5.8	-4.4	±42.3	46.8	±42.7	-8.4	±0.5	-95.0	±44.8	38.2	±42.7	-56.6	±3.9
	**CLR-Leu^8^OT-VTR1b**	-83.6	±10.8	-49.4	±34.4	89.8	±37.8	-7.9	±0.8	-133.0	±32.6	81.9	±37.7	-51.1	±5.3
**Marmoset**	**Pro^8^OT-OTR**	-94.4	±5.3	-17.0	±3.4	61.5	±5.9	-8.2	±0.2	-111.4	±2.8	53.3	±5.7	-58.1	±5
	**Pro^8^OT-VTR1a**	-98.6	±4	-17.7	±0.7	60.3	±0.8	-8.7	±0.1	-116.3	±4.6	51.6	±0.8	-64.7	±4.2
	**Pro^8^OT-VTR1b**	-88.7	±2.1	-12.6	±8.2	54.7	±10.3	-7.9	±0.3	-101.3	±8.8	46.8	±10.6	-54.5	±2.9
	**CLR-Pro^8^OT-OTR**	-97.1	±8	-18.9	±4.4	63.3	±6.4	-8.7	±0.2	-115.9	±12.3	54.6	±6.2	-61.3	±7.1
	**CLR-Pro^8^OT-VTR1a**	-94.0	±14.5	-18.6	±4.1	60.4	±9.4	-8.2	±0.6	-112.5	±18.7	52.2	±8.8	-60.4	±10.2
	**CLR-Pro^8^OT-VTR1b**	-95.8	±6.5	-27.3	±5.3	73.6	±10	-8.5	±0.3	-123.2	±11.7	65.1	±9.7	-58.0	±2.1

Δ: Complex − Receptor − Ligand.VDWAALS: Van der Waals energy.EEL: Electrostatic energy.EPB: Electrostatic solvation free energy calculated using the Poisson-Boltzmann equation. ESURF: Nonpolar component of the solvation energy. GGAS: Gas-phase energy. GSOLV: Solvation free energy.

The values are in kJ mol^-1^.

Note that an earlier simulation (Li et al., 2014), based on Molecular Mechanics/Generalized Born Surface Area (MM/GBSA) analysis rather than MM/PBSA, the authors found -15.4 kcal, which gives -64.4 kJ for the human complex Leu^8^OT-OTR, a value considered by the authors as a moderate binding affinity. Since our calculation (Leu^8^OT-OTR ΔG_TOTAL =_ -51.6±1.0 kJ mol^-1^) involves much longer simulations than theirs, there is a reasonable agreement between the values, at least considering the human Leu^8^OT-OTR complex. 

A per-residue energy contribution to the intermolecular interactions of the complexes was also calculated using the MM/PBSA method. Across Figures S5-S7, per-residue energy maps reveal a shared organizational principle: a small set of residues accounts for most of the favorable stabilization (blue), while intermittent unfavorable patches (red) occur at the interface. These simulations were performed with OT as the ligand, even for VTR1a and VTR1b, whose canonical hormone is VT. Red contributions reflect positive free energy and often mark contacts that are transient or repulsive; in ligand-receptor systems, such weaker interactions are expected and can facilitate reversibility and desensitization, so residues of functional relevance may legitimately appear in red.

Ortholog comparisons of OTR between human and *Callithrix* (Figure S5) revealed a strong conservation of hotspot residues, including Lys116, Phe175, Trp188, Ile201 and Phe311, yielding comparable per-residue energy profiles across species. The conservation of Lys116 is particularly intriguing, as its repulsive character (more intense reddish throughout the dynamics) may contribute to receptor desensitization by destabilizing the ligand-receptor interface. Such a mechanism would be consistent with the requirement for tight regulation of OT-receptor signaling, ensuring rapid disengagement after activation. The persistence of this residue across primate lineages suggests that maintaining a controlled balance between activation and desensitization represents an evolutionarily conserved feature of the oxytocinergic system. Cholesterol increases net stabilization (more intense blues) more clearly in OTR than in the vasopressin receptors, without changing which residues dominate binding. For VTR1a (Figure S6), hotspots such as Lys128, Phe189, Val217, Phe307, Gln311 e Ile330 are conserved across species; CLR produces modest amplification of favorable contributions, again preserving hotspot identity. For VTR1b (Figure S7), despite numbering differences (*e.g*., human Lys111/Phe172/Cys186/Trp187 *vs. Callithrix* Lys132/Phe195/Cys128), the functional equivalents are retained, indicating orthologous conservation. The CLR’s stabilizing effect is present but less pronounced than in OTR and comparable to VTR1a. The presence of a lysine residue at the orthologous position in the vasopressin receptors, consistently showing more intense reddish signals throughout the dynamics, indicates that this potential repulsive mechanism is preserved across oxytocinergic paralogs and conserved among primates.

Paralog comparisons within species (OTR *vs.* VTR1a *vs.* VTR1b) shows that, in humans, OTR exhibits a more concentrated band of strong favorable contributions (blue) at a few residues, and shows the largest cholesterol-driven stabilization among the three receptors. VTR1a and VTR1b display a more distributed interaction landscape with a mix of modest blues and localized reds, consistent with a broader set of weaker contacts that can aid kinetic flexibility. In *Callithrix jacchus*, the same pattern holds: OTR retains the clearest CLR responsiveness, while VTR1a/VTR1b show subtler modulation. Across all three receptors, hotspot identity is largely shared with the human counterparts, underscoring evolutionary maintenance of the binding-site architecture.

### Amino acids comparison

The alignment between the canonical OTR in *Homo sapiens* and *Callithrix jacchus* revealed that 24 out of 389 positions (6.17%) exhibited amino acid differences between the species, while 365 positions (93.83%) were conserved. Of the positions critical for interaction between the neurohormones and their canonical receptors, ten of them (96, 99, 295, 201, 175, 307, 116, 311, 186, 188) are shared between the species (Figure S8). These ten homologous positions showed no amino acid variation between *Homo sapiens* and *Callithrix jacchus*, suggesting significant functional conservation at these sites. Considering that 17 amino acids were identified as important for interaction in each species, these positions represent 58.82% of the critical interaction sites between the neurohormones and their canonical receptors.

Among the positions important for the interaction between the human OTR and Leu⁸OT, only position 197 differs between the two species: *Homo sapiens* has a proline (Pro), an apolar residue, whereas *Callithrix jacchus* has a serine (Ser), which is polar and capable of forming hydrophilic interactions. This difference may reflect structural variation within the oxytocinergic system between the two species, potentially associated with species-specific receptor-ligand interactions. However, further experimental studies are necessary to assess any functional consequences. On the other hand, none of the 17 critical positions for interaction between the *Callithrix jacchus* OTR and Pro^8^OT showed amino acid differences between the species, indicating strong evolutionary conservation.

The observed conservation at critical receptor-hormone interaction positions, especially the ten positions shared between the two species, suggests that these regions play a vital role in the function of OTR in primates, maintaining the stability and effectiveness of neurohormonal interaction. While the conservation at key interaction sites highlights their functional importance, the difference at position 197 may indicate a species-specific modulation of the interaction in *Homo sapiens*, potentially linked to physiological or behavioral traits. Nonetheless, *in vitro* and *in vivo* experimental validation is necessary to support this interpretation.

The alignment of VTR1a sequences revealed that 32 out of 418 amino acid positions (7.66%) varied between *Homo sapiens* and *Callithrix jacchus*, while 386 positions (92.34%) remained conserved. Among the positions critical for interaction between Leu^8^OT and Pro^8^OT and VTR1a receptors in each species, nine positions (128, 132, 330, 108, 307, 311, 217, 315, 189) were common between species (Figure S9). These nine homologous positions showed no amino acid variation, suggesting significant functional conservation at these sites.

Considering that *Homo sapiens* has 13 important positions for interaction, the proportion of identical positions between species is 69.23%. In *Callithrix jacchus*, with 17 important positions, the proportion of conserved positions is 52.94%. None of these 13 critical positions for interaction between *Homo sapiens* VTR1a and Leu^8^OT exhibited variation between species, indicating strong evolutionary conservation at these sites. Only one of the 17 critical positions for interaction between *Callithrix jacchus* VTR1a and Pro^8^OT, position 213, exhibited a different amino acid between species: *Homo sapiens* has serine (Ser), a polar amino acid, while *Callithrix jacchus* has proline (Pro), which is apolar, the exact opposite pattern observed in OTR. This finding can be reflecting specific modulation of interaction in *Callithrix jacchus*, possibly related to physiological or behavioral particularities of this species. However, any association with species-specific physiological or behavioral traits remains speculative and requires experimental validation.

The main structural differences between the VTR1b of *Homo sapiens* and *Callithrix jacchus* involve a deletion of four amino acids in the third intracellular loop of the VTR1b of *Callithrix jacchus*, which is also present in the other five New World primate species analyzed in the database, and: (*Saimiri boliviensis boliviensis*, *Aotus nancymaae*, *Cebus imitator*, *Sapajus apella*, and *Callithrix jacchus*). This deletion was described initially by Fam et al. (2019). Additionally, the presence of the genomes with higher coverage in the database considering the regions of interest allowed us to observe that *Callithrix jacchus* VTR1b has a 21-amino acid duplication in the N-terminal portion. This characteristic is shared only with *Sapajus apella* (XP_032144897.1) among all primate species with VTR1b protein sequences annotated in NCBI (as of a search conducted on 08/23/2024).

The total length of the alignment, considering the indels, is 445 positions, of which there is an amino acid difference between species in 64 positions (14.38%) considering indels and 39 positions (8.76%) considering only amino acid differences. The analysis of homologous positions between *Homo sapiens* and *Callithrix jacchus* VTR1b receptors reveals conservation of seven important positions for interactions with the neurohormone, with the same amino acid present in both species. This conservation suggests that these positions are critical for receptor function, reflecting an evolutionary need to maintain these interactions intact. Since *Homo sapiens* has 17 important positions for interaction, the proportion of important positions that are identical between species is 41.18%. *Callithrix jacchus*, in turn, has 13 important positions for interaction, so the important positions for this species represent 53.85%.

Although there are other positions unique to each species that may be linked to the potential functional variations, the high degree of conservation in the common positions highlights the importance of these regions for maintaining receptor function in different evolutionary contexts, at least considering primates.

In summary, the analysis suggests the existence of a conserved primate pattern that has been maintained throughout evolution. This pattern indicates that key positions critical for biological functions tend to remain conserved, thereby ensuring the essential and similar pleiotropic roles (*e.g*., in physiology and behavior) modulated by molecules of the oxytocinergic system in primates. Nevertheless, some notable differences were observed. These findings may also hold important implications for future studies on the evolution of neuroendocrine systems.

## Conclusion

The discovery of oxytocin variants in New World monkeys, particularly within the Cebidae, Callitrichidae, and Atelidae families, challenges the conventional notion of a universal oxytocin amino acid sequence among placental mammals.

Our exploration of ligand-receptor interactions through molecular modeling, docking, and molecular dynamics simulations provides, for the first time, valuable insights into the structural and functional characteristics of oxytocinergic molecules in *Homo sapiens* and *Callithrix jacchus*. Our analysis indicates that the Pro^8^OT form, present in the *Callithrix jacchus* species, appears to maintain a general pattern found in primates, with some specific properties and interactions with its canonical receptor (OTR), potentially influencing receptor reversibility/desensitization and downstream signaling pathways. Additionally, newly identified cholesterol interactions influence receptor binding and stability.

Furthermore, our MM/PBSA analysis reveals that the marmoset *Callithrix jacchus* complexes generally demonstrate more favorable interactions and binding energies between Pro^8^OT and their receptors compared to the human counterparts. These findings suggest the presence of distinct evolutionary adaptations within the oxytocinergic systems of these two primate species. 

Regarding the cholesterol role it is important to observe that the identity of key stabilizing residues is conserved across orthologs and largely consistent across paralogs; CRL modulates the magnitude (not the identity) of favorable interactions, with the strongest effect in OTR (both species) and subtler effects in VTR1a/VTR1b; unfavorable contributions at specific contacts are expected and can be functionally useful for reversibility and signal termination. Collectively, these patterns support an evolutionarily conserved binding interface in primates, with receptor-specific susceptibility to sterol-mediated stabilization under membrane-free conditions.

In summary, this research enhances our understanding of molecular pathways that may exhibit general patterns across primates, while also highlighting the differential functionality of taxon-specific OTs and their cognate OTRs within primate taxa. Further investigations are necessary to validate these findings and deepen our understanding of the intricate nuances of this fascinating system, as well as its functional role, including from an evolutionary perspective.

## Supplementary Material

The following online material is available for this article:

Table S1 - MM/PBSA binding free energies (kJ·mol⁻¹) for *Homo sapiens* and *Callithrix jacchus* oxytocin-receptor complexes without cholesterol.

Table S2 - MM/PBSA binding free energies (kJ·mol⁻¹) for *Homo sapiens* and *Callithrix jacchus* oxytocin-receptor complexes with cholesterol.

Table S3 - Interacting residues and interaction types in *Homo sapiens* complexes.

Table S4 - Interacting residues and interaction types in *Callithrix jacchus* complexes.

Table S5 - Interaction catalogue for cholesterol (CLR)-receptor complexes in *Homo sapiens* and *Callithrix jacchus.*

Figure S1 - Sites of cholesterol interactions with *Homo sapiens* and marmoset *Callithrix jacchus* OTR, VTR1a, and VTR1b receptors.

Figure S2 - Root Mean Square Deviation (RMSD) of *Homo sapiens* (Leu^8^OT) and marmoset *Callithrix jacchus* (Pro^8^OT) complexes (with and without cholesterol).

Figure S3 - Root Mean Square Fluctuation (RMSF) analysis of oxytocin complexes in *Homo sapiens* (Leu^8^OT) and marmoset *Callithrix jacchus* (Pro^8^OT), both with and without cholesterol.

Figure S4 - The average number of hydrogen bonds for *Homo sapiens* and marmoset *Callithrix jacchus* oxytocinergic complexes.

Figure S5 - Per-residue energy contributions to the formation of oxytocin-oxytocin receptor (OT-OTR) complexes and cholesterol-oxytocin-oxytocin receptor (CLR-OT-OTR) complexes in *Homo sapiens* (“Hum” in the figure) and *Callithrix jacchus* (“Cal” in the figure; marmoset).

Figure S6 - Per-residue energy contributions to the formation of oxytocin-vasotocin receptor 1a (OT-VTR1a) complexes and cholesterol-oxytocin-vasotocin receptor 1a (CLR-OT-VTR1a) complexes in *Homo sapiens* (“Hum” in the figure) and *Callithrix jacchus* (“Cal” in the figure; marmoset).

Figure S7 - Per-residue energy contributions to the formation of oxytocin-vasotocin receptor 1b (OT-VTR1b) complexes and cholesterol-oxytocin-vasotocin receptor 1b (CLR-OT-VTR1b) complexes in *Homo sapiens* (“Hum” in the figure) and *Callithrix jacchus* (“Cal” in the figure; marmoset).

Figure S8 - The first two figures show the *Homo sapiens* OTR and the important sites for interaction with Leu^8^OT.

Figure S9 - The first two figures show the *Homo sapiens* VTR1a receptor and the important sites for interaction with Leu^8^OT.

Figure S10 - The first two figures show the *Homo sapiens* VTR1b and the important sites for interaction with Leu^8^OT.

## Data Availability

 The datasets underlying the results presented in the next section are available from the corresponding author upon reasonable request. Input files required to reproduce the molecular dynamics simulations will be provided for academic research purposes upon request.
