# Supplementary material for: Computational modeling of oxytocin-receptors interactions with the common marmoset Callithrix jacchus Pro8OT variant
Source: Genet Mol Biol. 2025 Dec 1;48(4):e20250058. doi: 10.1590/1678-4685-GMB-2025-0058 (PMC12704488; doi:10.1590/1678-4685-GMB-2025-0058)
Supplement: Table S1 - [file 1415-4757-GMB-48-04-e20250058-s1.pdf]

Genetics and Molecular Biology

https://doi.org/10.1590/1678-4685-GMB-2025-0058

Supplementary Material to "Computational modeling of oxytocin-receptors interactions with the common marmoset *Callithrix jacchus* Pro⁸OT variant"

Table S1- Binding Free Energy MM/PBSA (in kJ mol^{-1}) computations of *Homo sapiens* and marmoset *Callithrix jacchus* oxytocin and cognate receptors complexes without cholesterol.

Organism	System	R.	ΔEVDWAALS	ΔEEL	ΔEPB	ΔESURF	ΔGGAS	ΔGSOLV	ΔGTOTAL
Human	**Leu^{8}OT-OTR**	1	-87.6	63.8	-19.3	8.2	-23.8	-27.4	**-51.3**
		2	-86.7	27.4	17.2	-8.6	-59.3	8.7	**-50.7**
		3	-90.5	64.7	-18.4	-8.6	-25.8	-26.9	**-52.7**
	**Leu^{8}OT-VTR1a**	1	-97.0	-76.9	121.7	-8.6	-173.9	113.1	**-60.8**
		2	-94.7	-31.7	75.6	-8.3	-126.3	67.3	**-59.0**
		3	-84.1	-20.4	63.4	-8.0	-104.4	55.4	**-49.1**
	**Leu^{8}OT-VTR1b**	1	-65.2	35.6	4.1	-7.2	-29.6	-3.0	**-32.6**
		2	-69.8	-10	47.7	-7.0	-79.8	40.7	**-39.1**
		3	-76.2	38.5	3.1	-7.3	-37.7	-4.2	**-41.9**
Marmoset	**Pro^{8}OT-OTR**	1	-89.1	-19.1	58.9	-8.2	-108.2	50.7	**-57.5**
		2	-94.4	-18.8	68.2	-8.4	-113.2	59.8	**-53.4**
		3	-99.7	-13.1	57.4	-8.0	-112.8	49.4	**-63.4**
	**Pro^{8}OT-VTR1a**	1	-94.2	-17	59.8	-8.8	-111.2	51.0	**-60.2**
		2	-102	-17.9	59.9	-8.6	-119.9	51.4	**-68.5**
		3	-99.7	-18.3	61.2	-8.7	-117.9	52.5	**-65.4**
	**Pro^{8}OT-VTR1b**	1	-88.7	-22	66.5	-7.6	-110.7	58.9	**-51.8**
		2	-90.7	-9.2	50.4	-8.1	-99.9	42.3	**-57.6**
		3	-86.6	-6.7	47.3	-8.1	-93.2	39.2	**-54.0**

Δ: Complex (Receptor−Ligand). VDWAALS: Van der Waals. eeEEL: Electrostatic energy. EGB: electrostatic solvation free energy evaluated from the generalized Born equation. ESURF: the nonpolar component of the solvation energy. GGAS: gas-phase energy. GSOLV: solvation free energy. The values are in kJ mol^{-1}.
