# Supplementary material for: Computational modeling of oxytocin-receptors interactions with the common marmoset Callithrix jacchus Pro8OT variant
Source: Genet Mol Biol. 2025 Dec 1;48(4):e20250058. doi: 10.1590/1678-4685-GMB-2025-0058 (PMC12704488; doi:10.1590/1678-4685-GMB-2025-0058)
Supplement: Table S2 - [file 1415-4757-GMB-48-04-e20250058-s2.pdf]

Genetics and Molecular Biology

https://doi.org/10.1590/1678-4685-GMB-2025-0058

Supplementary Material to "Computational modeling of oxytocin-receptors interactions with the common marmoset *Callithrix jacchus* Pro⁸OT variant"

Table S2 - Binding Free Energy MM/PBSA (in kJ mol^{-1}) computations of *Homo sapiens* and marmoset *Callithrix jacchus* oxytocin and cognate receptors complexes with cholesterol.

Organism	System	R.	ΔE$_{VDWAALS}$	ΔE$_{EL}$	ΔE$_{PB}$	ΔE$_{SURF}$	ΔG$_{GAS}$	ΔG$_{SOLV}$	ΔG$_{TOTAL}$
Human	**CLR-Leu^{8}OT-OTR**	1	-93.6	36.5	9	-8.8	-57.1	0.3	**-56.9**
		2	-81.7	36.4	7.7	-8.2	-45.3	-0.5	**-45.8**
		3	-83.4	38	6.2	-8.1	-45.4	-1.9	**-47.3**
	**CLR-Leu^{8}OT-VTR1a**	1	-86.9	43.7	-2.2	-8.4	-43.3	-10.7	**-53.9**
		2	-87.5	-35.6	76.2	-7.9	-123.13	68.2	**-54.9**
		3	-97.2	-21.3	66.3	-8.9	-118.6	57.2	**-61.1**
	**CLR-Leu^{8}OT-VTR1b**	1	-93.7	-61	114	-8.7	-154.7	105.3	**-49.4**
		2	-72.2	-76.5	109.1	-7.2	-148.8	101.9	**-46.9**
		3	-84.8	-10.7	46.2	-7.9	-95.5	38.4	**-57.1**
Marmoset	**CRL-Pro^{8}OT-OTR**	1	-92.1	-15.8	61.7	-8.6	-107.9	53.1	**-54.8**
		2	-92.8	-16.9	57.9	-8.5	-109.7	49.3	**-60.3**
		3	-106.3	-23.9	70.3	-8.9	-130.1	61.4	**-68.8**
	**CRL-Pro^{8}OT-VTR1a**	1	-105.8	-22.1	66.4	-8.5	-127.9	57.9	**-70.1**
		2	-98.3	-19.6	65.2	-8.6	-117.9	56.6	**-61.4**
		3	-77.8	-14	49.6	-7.6	-91.7	42	**-49.8**
	**CRL-Pro^{8}OT-VTR1b**	1	-102	-30.7	81.2	-8.8	-132.7	72.4	**-60.3**
		2	-89	-21.2	62.3	-8.3	-110.1	54.1	**-56.1**
		3	-96.5	-30.1	77.4	-8.5	-126.7	68.9	**-57.7**

Δ: Complex (Receptor−Ligand). VDWAALS: Van der Waals. EEL:Electrostatic energy. EGB: electrostatic solvation free energy evaluated from the generalized Born equation. ESURF: the nonpolar component of the solvation energy. GGAS: gas-phase energy. GSOLV: solvation free energy. The values are in kJ mol^{-1}.
