# Supplementary material for: Computational modeling of oxytocin-receptors interactions with the common marmoset Callithrix jacchus Pro8OT variant
Source: Genet Mol Biol. 2025 Dec 1;48(4):e20250058. doi: 10.1590/1678-4685-GMB-2025-0058 (PMC12704488; doi:10.1590/1678-4685-GMB-2025-0058)
Supplement: Table S3 - [file 1415-4757-GMB-48-04-e20250058-s3.pdf]

Genetics and Molecular Biology

https://doi.org/10.1590/1678-4685-GMB-2025-0058

Supplementary Material to "Computational modeling of oxytocin-receptors interactions with the common marmoset *Callithrix jacchus* Pro^{8}OT variant"

Table S3 - *Homo sapiens* complexes, interacting residues and type of interactions.

Complex	Receptor's residue	Interaction
Leu^{8}OT-OTR	GLN92	Conventional Hydrogen Bond
	TRP99	Alkyl
	ILE201	Pi-alkyl/Alkyl
	PHE311	Pi-sulfur
Leu^{8}OT-VTR1a	ALA205	Alkyl
	VAL217	Alkyl/Carbon Hydrogen Bond
	GLN311	Conventional Hydrogen Bond
	TRP322	Conventional Hydrogen Bond/Pi-sigma
	ILE330	Pi-Alkyl
	THR333	Conventional Hydrogen Bond
Leu^{8}OT-VTR1b	ALA188	Conventional Hydrogen Bond
	PHE320	Pi-Sulfur/Pi-Alkyl
CLR-Leu^{8}OT-OTR	PRO95	Carbon Hydrogen Bond/Alkyl
	TRP99	Alkyl
	LYS116	Conventional Hydrogen Bond
CLR-Leu^{8}OT-VTR1a	TRP111	Pi-pi T-Shaped/Alkyl
	GLN311	Conventional Hydrogen Bond
	SER314	Conventional Hydrogen Bond/Carbon Hydrogen Bond
	ILE330	Pi-alkyl
	ALA334	Pi-alkyl/alkyl
CLR-Leu^{8}OT-VTR1b	GLN168	Conventional Hydrogen Bond
	ALA188	Conventional Hydrogen Bond/Alkyl
	PRO196	Alkyl
