# Supplementary material for: Computational modeling of oxytocin-receptors interactions with the common marmoset Callithrix jacchus Pro8OT variant
Source: Genet Mol Biol. 2025 Dec 1;48(4):e20250058. doi: 10.1590/1678-4685-GMB-2025-0058 (PMC12704488; doi:10.1590/1678-4685-GMB-2025-0058)
Supplement: Table S4 - [file 1415-4757-GMB-48-04-e20250058-s4.pdf]

Genetics and Molecular Biology

https://doi.org/10.1590/1678-4685-GMB-2025-0058

Supplementary Material to "Computational modeling of oxytocin-receptors interactions with the common marmoset *Callithrix jacchus* Pro^{8}OT variant"

Table S4 - Marmoset *Callithrix jacchus* complexes, interacting residues and type of interactions.

Complex	Receptor's residue	Interaction
Pro^{8}OT-OTR	GLN92	Conventional Hydrogen Bond/Carbon Hydrogen Bond
	TRP99	Carbon Hydrogen Bond/Alkyl
	GLN119	Conventional Hydrogen Bond
	VAL120	Alkyl
	ILE204	Alkyl
	GLN295	Conventional Hydrogen Bond
	SER298	Carbon Hydrogen Bond
	LYS306	Conventional Hydrogen Bond
	MET315	Conventional Hydrogen Bond/Pi-alkyl
Pro^{8}OT-VTR1a	GLN104	Conventional Hydrogen Bond
	GLN108	Conventional Hydrogen Bond
	GLN131	Conventional Hydrogen Bond
	VAL217	Alkyl
	PHE308	Carbon Hydrogen Bond/Alkyl
	GLN311	Conventional Hydrogen Bond
	VAL315	Pi-alkyl/Alkyl
	ILE330	Conventional Hydrogen Bond/Alkyl
Pro^{8}OT-VTR1b	TRP115	Conventional Hydrogen Bond/Alkyl
	TRP208	Alkyl/Pi-sigma
	PRO217	Conventional Hydrogen Bond/Alkyl/Pi-alkyl
	GLN318	Conventional Hydrogen Bond
	SER337	Conventional Hydrogen Bond/Alkyl
CLR-Pro^{8}OT-OTR	TRP99	Conventional Hydrogen Bond/Pi-pi stacked/Pi-alkyl
	LYS116	Conventional Hydrogen Bond/Alkyl
	ILE204	Alkyl
	GLN295	Conventional Hydrogen Bond
	PHE311	Pi-Sulfur/Pi-donor Hydrogen Bond
	ILE312	Carbon Hydrogen Bond/Alkyl
	MET315	Alkyl
CLR-Pro^{8}OT-VTR1a	GLN108	Conventional Hydrogen Bond
	LYS128	Conventional Hydrogen Bond
	GLN131	Conventional Hydrogen Bond
	PRO213	Pi-alkyl
	VAL217	Alkyl

Complex	Receptor's residue	Interaction
	MET220	Conventional Hydrogen Bond/Alkyl
	GLN311	Conventional Hydrogen Bond
	TRP322	Pi-pi T-Shaped/Carbon Hydrogen Bond
	ILE330	Alkyl
CLR-Pro^{8}OT-VTR1b	GLN112	Carbon Hydrogen Bond
	TRP115	Conventional Hydrogen Bond/Carbon Hydrogen Bond
	HIS329	Alkyl/Pi-alkyl
	THR333	Carbon Hydrogen Bond
