# Supplementary material for: Computational modeling of oxytocin-receptors interactions with the common marmoset Callithrix jacchus Pro8OT variant
Source: Genet Mol Biol. 2025 Dec 1;48(4):e20250058. doi: 10.1590/1678-4685-GMB-2025-0058 (PMC12704488; doi:10.1590/1678-4685-GMB-2025-0058)
Supplement: Table S5 - [file 1415-4757-GMB-48-04-e20250058-s5.pdf]

Genetics and Molecular Biology

https://doi.org/10.1590/1678-4685-GMB-2025-0058

Supplementary Material to “Computational modeling of oxytocin-receptors interactions with the common marmoset *Callithrix jacchus* Pro^{8}OT variant”

Table S5 - List of interactions observed in simulations of *Homo sapiens* and marmoset *Callithrix jacchus* cholesterol (CLR) -receptor complexes.

Organism	Complex	Receptor's residue	Interaction	Cholesterol moiety
Human	CLR-OTR	PRO170	van der Waals	aliphatic moiety
		HIS173	Carbon hydrogen bond	tetracyclic moiety
		ILE192	Alkyl, pi-alkyl	tetracyclic moiety
		TRP195	Alkyl, pi-alkyl	tetracyclic moiety
		GLY196	van der Waals	tetracyclic moiety
		ALA199	Alkyl, pi-alkyl	tetracyclic moiety
		TYR200	van der Waals	tetracyclic moiety
		TRP203	Pi-sigma	aliphatic moiety
	CLR-VTR1a	PRO184	Alkyl, pi-alkyl	tetracyclic moiety
		VAL188	Alkyl, pi-alkyl	tetracyclic moiety
		THR206	Conventional hydrogen bond	tetracyclic moiety
		ILE208	Alkyl, pi-alkyl	tetracyclic moiety
		TRP211	Alkyl, pi-alkyl	tetracyclic moiety
		ALA215	Alkyl, pi-alkyl	tetracyclic moiety
		TYR216	Alkyl, pi-alkyl	tetracyclic moiety
		TRP219	Alkyl, pi-alkyl	aliphatic moiety
	CLR-VTR1b	PRO167	Alkyl, pi-alkyl	tetracyclic moiety
		ILE171	Alkyl, pi-alkyl	tetracyclic moiety
		ASP189	Conventional hydrogen bond	tetracyclic moiety
		TRP194	Alkyl, pi-alkyl	tetracyclic moiety
		ALA198	Alkyl, pi-alkyl	tetracyclic moiety
		TYR199	Alkyl, pi-alkyl	tetracyclic moiety
		TRP202	Alkyl, pi-alkyl	both
Marmoset	CLR-OTR	PRO170	Alkyl, pi-alkyl	tetracyclic moiety
		HIS173	Carbon hydrogen bond	tetracyclic moiety
		ILE174	Alkyl, pi-alkyl	tetracyclic moiety
		ILE192	Alkyl, pi-alkyl	tetracyclic moiety
		TRP195	Alkyl, pi-alkyl	tetracyclic moiety
		ALA199	Alkyl, pi-alkyl	tetracyclic moiety
		TYR200	Alkyl, pi-alkyl	tetracyclic moiety
		TRP203	Alkyl, pi-alkyl	aliphatic moiety
	CLR-VTR1a	PRO184	Alkyl, pi-alkyl	tetracyclic moiety
		VAL188	Alkyl, pi-alkyl	aliphatic moiety

Organism	Complex	Receptor's residue	Interaction	Cholesterol moiety
		THR206	van der Waals	aliphatic moiety
		PHE207	Alkyl, pi-alkyl	tetracyclic moiety
		ILE208	van der Waals	tetracyclic moiety
		TRP211	van der Waals	tetracyclic moiety
		GLY212	van der Waals	aliphatic moiety
		PRO213	van der Waals	tetracyclic moiety
		ALA215	Alkyl, pi-alkyl	tetracyclic moiety
		TYR216	van der Waals	tetracyclic moiety
		TRP219	Pi-sigma	tetracyclic moiety
	CLR-VTR1b	LEU185	Alkyl, pi-alkyl	tetracyclic moiety
		PRO188	Alkyl, pi-alkyl	tetracyclic moiety
		ILE192	Alkyl, pi-alkyl	aliphatic moiety
		PHE211	Alkyl, pi-alkyl	aliphatic moiety
		VAL219	Alkyl, pi-alkyl	aliphatic moiety
		TYR220	Alkyl, pi-alkyl	both
		TRP223	Alkyl, pi-alkyl	both
