# Supplementary material for: Computational modeling of oxytocin-receptors interactions with the common marmoset Callithrix jacchus Pro8OT variant
Source: Genet Mol Biol. 2025 Dec 1;48(4):e20250058. doi: 10.1590/1678-4685-GMB-2025-0058 (PMC12704488; doi:10.1590/1678-4685-GMB-2025-0058)
Supplement: Figure S1 - [file 1415-4757-GMB-48-04-e20250058-s6.pdf]

https://doi.org/10.1590/1678-4685-GMB-2025-0058

Supplementary Material to "Computational modeling of oxytocin-receptors interactions with the common marmoset *Callithrix jacchus* Pro^{8}OT variant"

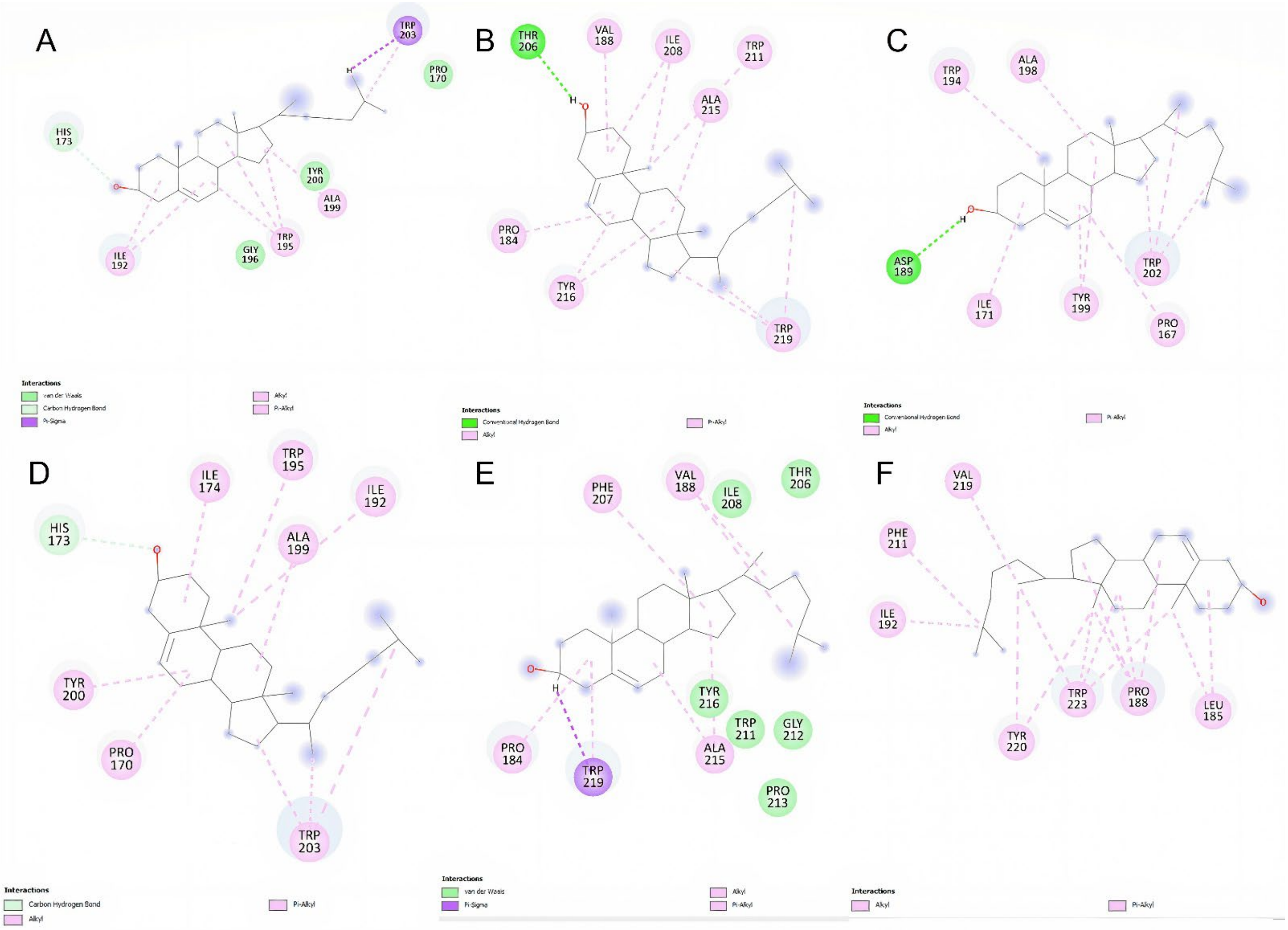

Figure S1 - Sites of cholesterol interactions with *Homo sapiens* and marmoset *Callithrix jacchus* OTR, VTR1a, and VTR1b receptors. A) Human CLR-OTR complex; B) Human CLR-VTR1a complex; C) Human C-VTR1b complex; D) Marmoset CLR-OTR complex; E) Marmoset CLR-VTR1a complex; F) Marmoset CLR-VTR1b complex. Light pink: alkyl and pi-alkyl interactions; purple: pi-sigma interactions; light green: Van der Waals and carbon-hydrogen bonds; green: conventional hydrogen bonds.
