# Supplementary material for: Computational modeling of oxytocin-receptors interactions with the common marmoset Callithrix jacchus Pro8OT variant
Source: Genet Mol Biol. 2025 Dec 1;48(4):e20250058. doi: 10.1590/1678-4685-GMB-2025-0058 (PMC12704488; doi:10.1590/1678-4685-GMB-2025-0058)
Supplement: Figure S3 - [file 1415-4757-GMB-48-04-e20250058-s8.pdf]

Genetics and Molecular Biology

https://doi.org/10.1590/1678-4685-GMB-2025-0058

Supplementary Material to "Computational modeling of oxytocin-receptors interactions with the common marmoset *Callithrix jacchus* Pro⁸OT variant"

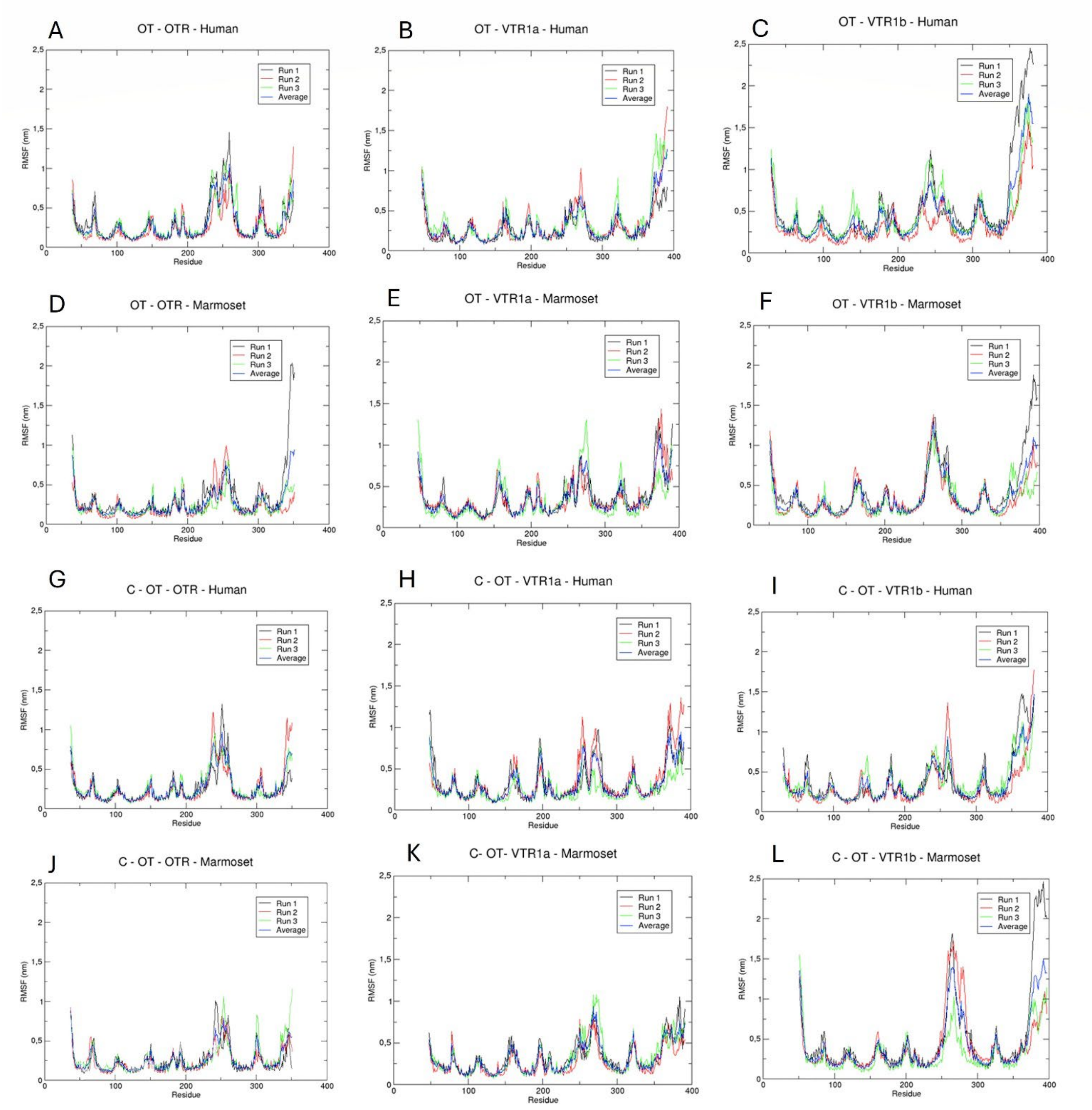

Figure S3 - Root Mean Square Fluctuation (RMSF) analysis of oxytocin complexes in *Homo sapiens* (Leu^{8}OT) and marmoset *Callithrix jacchus* (Pro^{8}OT), both with and without cholesterol. The figure dis-plays three runs for each complex (represented by black, red, and green lines) along with the average of these runs (represented by the blue line).
