# Supplementary material for: Computational modeling of oxytocin-receptors interactions with the common marmoset Callithrix jacchus Pro8OT variant
Source: Genet Mol Biol. 2025 Dec 1;48(4):e20250058. doi: 10.1590/1678-4685-GMB-2025-0058 (PMC12704488; doi:10.1590/1678-4685-GMB-2025-0058)
Supplement: Figure S7 - [file 1415-4757-GMB-48-04-e20250058-s12.pdf]

Genetics and Molecular Biology

https://doi.org/10.1590/1678-4685-GMB-2025-0058

Supplementary Material to "Computational modeling of oxytocin-receptors interactions with the common marmoset *Callithrix jacchus* Pro[8]OT variant"

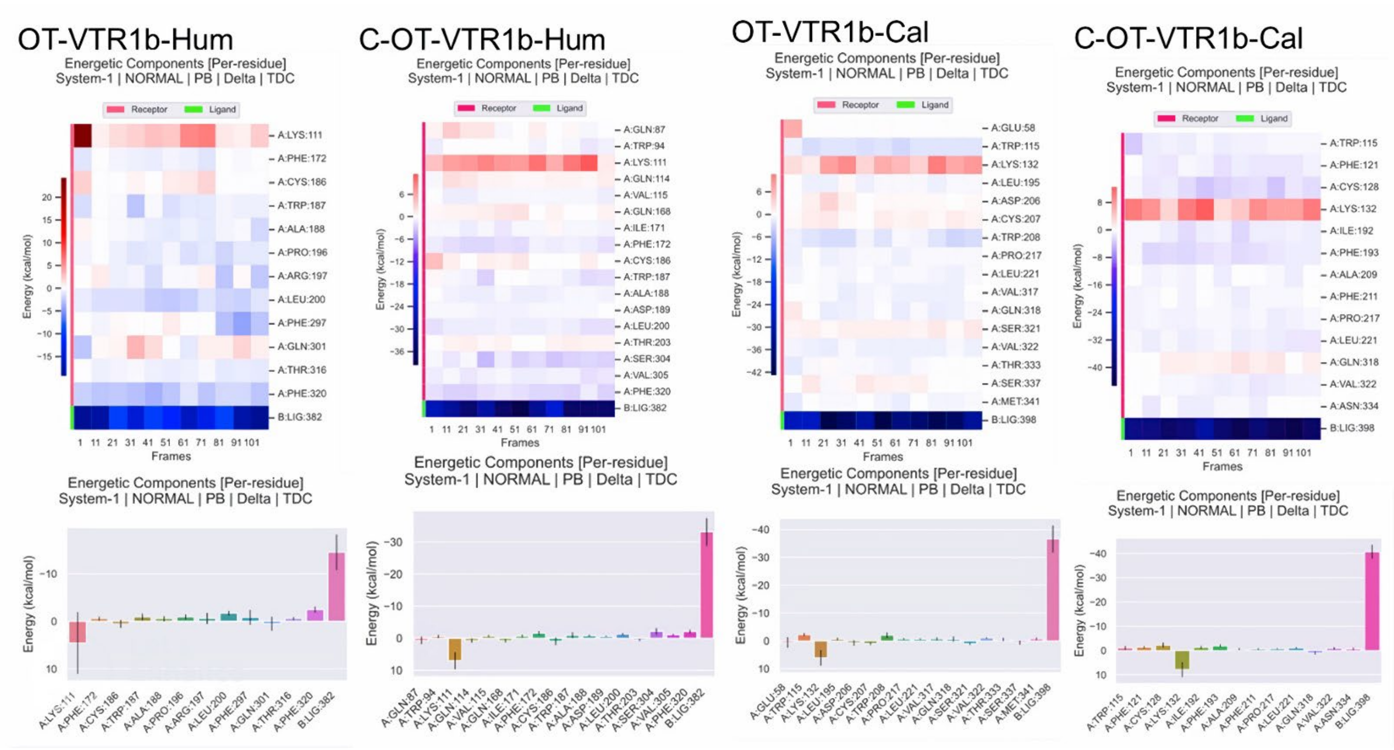

Figure S7 - Per-residue energy contributions to the formation of oxytocin–vasotocin receptor 1b (OT–VTR1b) complexes and cholesterol–oxytocin–vasotocin receptor 1b (CLR–OT–VTR1b) complexes in *Homo sapiens* ("Hum" in the figure) and *Callithrix jacchus* ("Cal" in the figure; marmoset). In the figure panels, "C" preceding OT–VTR1b denotes the cholesterol-bound complex. Heatmaps (top) display the energetic contribution (kcal/mol) of individual residues over simulation frames, with blue indicating favorable interactions and red indicating unfavorable interactions. Bar plots (bottom) summarize the average energy contribution per residue across the simulation. Residues from the receptor are shown in pink and from the ligand in green.
