# Supplementary material for: Computational modeling of oxytocin-receptors interactions with the common marmoset Callithrix jacchus Pro8OT variant
Source: Genet Mol Biol. 2025 Dec 1;48(4):e20250058. doi: 10.1590/1678-4685-GMB-2025-0058 (PMC12704488; doi:10.1590/1678-4685-GMB-2025-0058)
Supplement: Figure S9 - [file 1415-4757-GMB-48-04-e20250058-s14.pdf]

Genetics and Molecular Biology

https://doi.org/10.1590/1678-4685-GMB-2025-0058

Supplementary Material to "Computational modeling of oxytocin-receptors interactions with the common marmoset *Callithrix jacchus* Pro⁸OT variant"

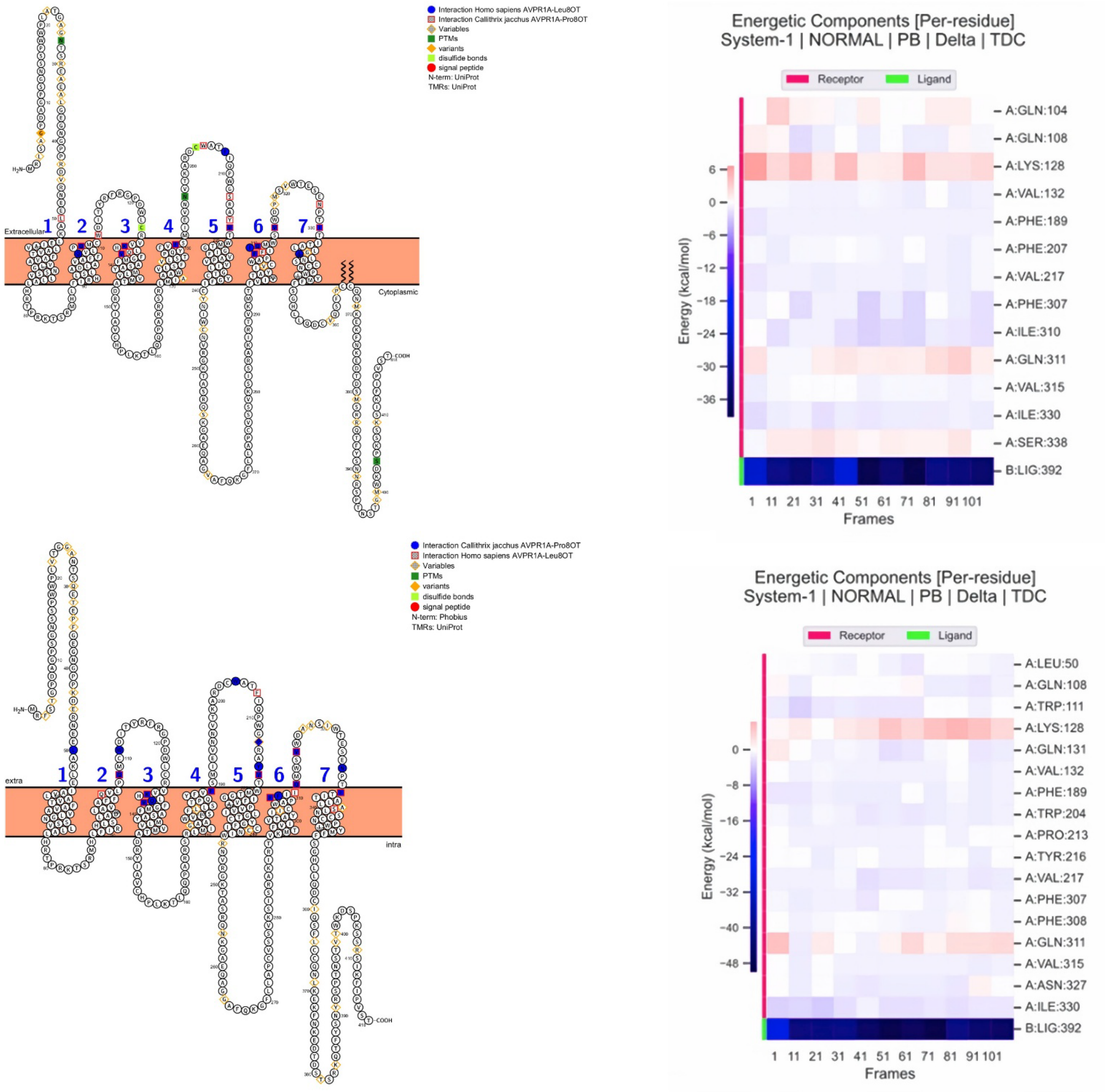

Figure S9 - The first two figures show the *Homo sapiens* VTR1a receptor and the important sites for interaction with Leu⁸OT. The figures in the bottom part show the *Callithrix jacchus* VTR1a and the important sites for interaction with Pro⁸OT.
